# Increased contralateral dynamic valgus in female athletes following ACL reconstruction

**DOI:** 10.1002/ksa.70291

**Published:** 2026-01-19

**Authors:** Fredrik Gaugg, Sebastian Bierke, Tilman Hees, Kerstin Siemßen, Bernd Wolfarth, Wolf Petersen

**Affiliations:** ^1^ Department for Orthopaedic Surgery and Traumatology Martin Luther Hospital Berlin Germany; ^2^ Department of Sports Medicine Humboldt University and Charité University Medicine Berlin Germany

**Keywords:** ACL injury, anterior cruciate ligament, gender differences, knee, prevention

## Abstract

**Purpose:**

Female athletes have a higher risk of anterior cruciate ligament (ACL) injury, which has been associated with neuromuscular deficits and dynamic valgus, while contralateral risk profiles after ACL reconstruction remain insufficiently characterized. The purpose of this study was to investigate whether female patients after ACL reconstruction exhibit greater contralateral valgus angles compared with male patients, indicating a higher risk profile for secondary ACL injury. It was hypothesized that dynamic valgus would be more pronounced in women than in men.

**Methods:**

In this predefined subanalysis of a controlled laboratory study, 26 patients (17 male and 9 female) were analysed approximately eight months after primary ACL reconstruction with either semitendinosus or quadriceps tendon autograft. Isokinetic strength testing and three‐dimensional (3D) motion capture analysis were performed. Participants executed vertical drop jumps and single‐leg jumps for distance. The primary outcome was the maximal contralateral knee valgus angle; secondary outcomes included hip and knee flexion angles, valgus moments and hamstrings‐to‐quadriceps (H/Q) ratio. Mann–Whitney *U* test was used for testing between male and female patients after testing for parametric distribution using Shapiro–Wilk test.

**Results:**

In the vertical drop jump, females demonstrated a significantly larger median maximal valgus angle compared to males (−2.9° [valgus] [interquartile range, IQR (−9.8) to 2.0] vs. 6.7° [varus] [IQR (−0.2) to 8.5], *p* = 0.016). No significant sex‐specific differences were observed in secondary kinematic parameters, single‐leg jump performance or isokinetic strength measures.

**Conclusion:**

Female patients exhibited greater contralateral dynamic valgus during drop jump landings after ACL reconstruction, suggesting sex‐specific neuromuscular risk patterns. These findings support the need for individualized rehabilitation and prevention strategies with a focus on dynamic valgus in female athletes.

**Level of Evidence:**

Level III.

**Abbreviation**
ACL: anterior cruciate ligament

## INTRODUCTION

Next to certain high‐risk sports such as soccer, basketball or handball [15, 29, 35] and anatomical conditions [1, 5, 34], current literature identifies female gender and neuromuscular factors as significant risk factors for an anterior cruciate ligament (ACL) rupture [12, 30, 34].

These include neuromuscular asymmetries, ‘quadriceps dominance’, weakness of the ischiocrural muscles and ‘dynamic valgus’ with reduced control of the hip and trunk muscles [1, 16, 17, 42]. As neuromuscular risk factors are potentially trainable factors, they are of particular relevance in the context of possible prevention programmes [34].

Quadriceps dominance refers to a muscular activation pattern in which the quadriceps femoris muscle is primarily used for stabilization [16]. This is reflected in faster quadriceps activation [21] and in reduced hamstring compared to quadriceps strength, commonly expressed as hamstrings‐quadriceps ratio (H/Q ratio). This has been identified as a risk factor for ACL rupture [31, 42].

At‐risk athletes show movement patterns similar to typical ACL injury mechanisms, landing after jumps with limited knee flexion and a valgus position, while the centre of gravity shifts posterior to the knee joint [16, 34]. The resulting functional valgus position is referred to as dynamic valgus [1, 16, 34]. The dynamic valgus position is associated with greater knee abduction moments [10, 17, 24, 32]. Particularly in combination with low knee flexion, it puts the ACL under high tension and has been shown to be a predictor of ACL rupture [2, 10].

These neuromuscular risk factors are predominantly observed in female athletes [16]. In movement analyses of various jump tests (stop jumps, drop jumps, single‐leg jumps), women landed with both, less bent legs and greater dynamic valgus compared to men [9, 20, 28]. Quadriceps dominance also appears to primarily affect women [16, 21]. Neuromuscular intervention programmes have been shown to have a protective effect in primary prevention [19].

Regarding secondary prevention of ACL rupture, there are significantly fewer studies in the literature. However, the risk of secondary rupture is known to be increased after ACL reconstruction, particularly on the contralateral side [45, 47]. Furthermore, data show that neuromuscular deficits persist for up to 5 years postoperatively after autologous ACL reconstruction [36].

The aim of this study is to investigate whether women exhibit a greater contralateral functional valgus position. The hypothesis is that dynamic valgus is more pronounced in women than in men after ACL reconstruction.

## MATERIALS AND METHODS

### Study design

This subanalysis of a controlled laboratory study is based on the same cohort as previously described by Gaugg et al. [11] and examined patients after reconstruction of the ACL with video motion analysis with regard to the occurrence of dynamic valgus. The identical raw three‐dimensional (3D) motion‐analysis and isokinetic strength data were reanalysed with a new grouping approach (male vs. female participants), focusing exclusively on the contralateral, non‐operated limb. No additional data were collected.

The methodology of the present study, including patient recruitment, surgical procedures, rehabilitation protocol, isokinetic strength testing and 3D motion analysis, were identical to the previous publication and are summarized below.

In addition to the primary research question of the original study, a predefined secondary hypothesis was formulated during the study planning, stating that female patients after ACL reconstruction would demonstrate a higher prevalence of risk movement patterns compared to male patients. The present work, therefore, represents a pre‐planned subanalysis addressing this secondary hypothesis.

In brief, patients who underwent primary ACL reconstruction with either a semitendinosus or quadriceps tendon graft at Martin Luther Hospital, Berlin, Germany, between November 2020 and July 2021 were included. Male and female patients from 18 to 40 years who underwent isolated primary anatomical ACL reconstruction with semitendinosus graft or quadriceps tendon graft were included. Patients with Revision ACL reconstruction and reconstruction using an allograft or patellar tendon autograft were excluded from this study, as well as all patients with an extension deficit of more than 5° in comparison to the contralateral side or an associated surgery such as high tibial osteotomy or posterior cruciate ligament surgery. Patients underwent functional assessment approximately 8 months postoperatively.

Participants were grouped according to sex to investigate potential gender‐specific differences in biomechanical parameters.

### Preliminary examination

Prior to admission to the motion analysis, all subjects underwent a clinical (e.g., Lachman test, pivot shift test, KT‐1000 measurement) and sonographic examination of the knee to assess joint effusions in order to subsequently grant approval for the following examinations based on the International Knee Documentation Committee (IKDC) scheme at IKDC A or B [22].

### Isokinetic force measurements

**Figure 1:**
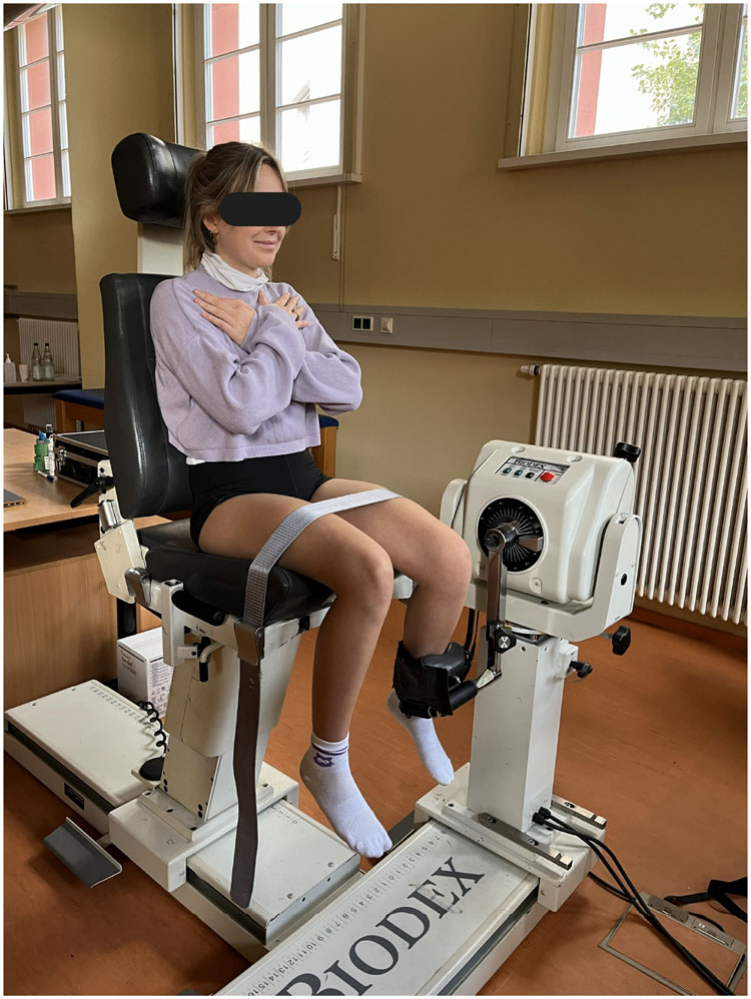
Experimental setup for isokinetic knee strength testing using a Biodex dynamometer. Participants were seated with the trunk and pelvis stabilized by straps. Knee flexion and extension strength were assessed at an angular velocity of 60°/s.

Isokinetic strength testing was conducted using the Biodex System 3 dynamometer (Biodex Medical Systems) at an angular velocity of 60°/s. After a standardized warm‐up, knee flexion and extension torques were assessed in a seated position with the hip flexed at 90° (see Figure 1; [7]). Measurements were initiated with the non‐operated side.

### Motion analysis

The motion analyses were carried out in the motion analysis laboratory of the Humboldt University of Berlin, Germany.

Ten 250 Hz infrared cameras, two BP400600‐2000 force plates (Advanced Medical Technology, Inc.) and the Vicon Motion Capture System Vicon Nexus 1.8.5 (Vicon Motion Systems Ltd) were used for this study. The motion analysis was carried out using the ‘Plug‐in Gait lower body’ model from Vicon [44].

**Figure 2:**
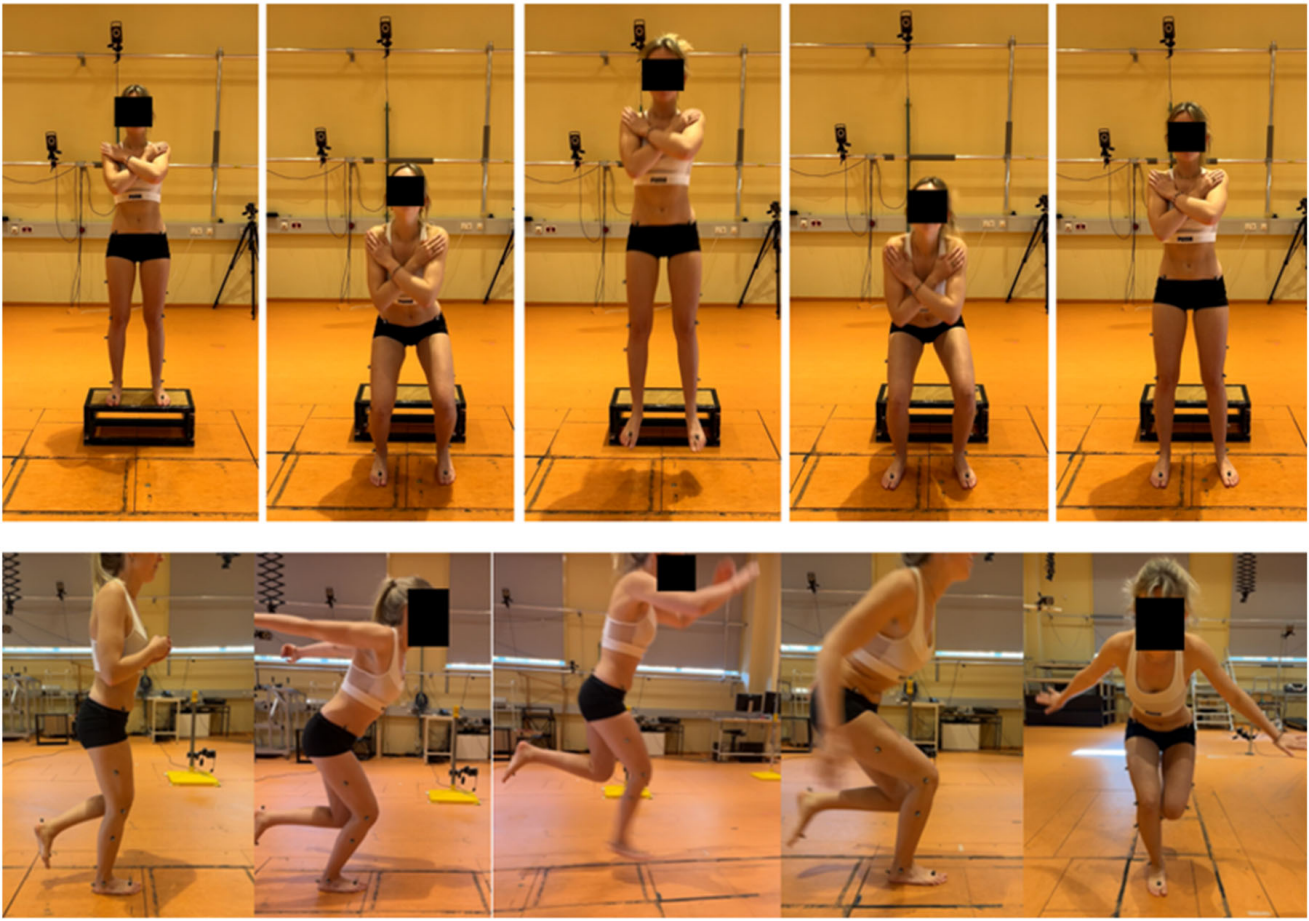
Illustration of the vertical drop jump test and the single‐leg jump for distance performed during 3D motion analysis. Reflective markers were attached according to the Vicon Plug‐in Gait lower body model (modified from Gaugg et al. [11]).

Participants performed a vertical drop jump and a single‐leg jump for distance, each repeated three times per leg (see Figure 2). All jumps were performed barefoot with standardized instructions.

### Data analysis

The primary outcome of this study was the maximum knee valgus angle in the drop jump and single‐leg jump test. The secondary outcomes were knee, hip flexion angles in both jump tests, as well as knee flexion and extension torque. Kinematic and kinetic data were extracted from Vicon Nexus and further processed in MATLAB (MathWorks). All measurements were reviewed for plausibility and transferred to an Excel spreadsheet (Microsoft Corporation) for statistical analysis.

The quantitative variables were analysed as continuous variables without categorization. Raw values were used. Group comparisons were performed between sex‐based groups (male vs. female). There were no missing data.

### Statistical analyses

The statistical analysis was performed with SPSS Statistics 27 (IBM). For the sample size calculation, a mean difference of 8° in maximum valgus angle (primary endpoint) was defined as clinically relevant, with an assumed standard deviation of 6.5° based on previously published data [14, 17]. The significance threshold was set at *p* < 0.05. Under these assumptions, a minimum of 11 participants per group would have been required to detect such a difference with 80% statistical power. This number was not achieved in the female group in this sub‐analysis.

Continuous baseline variables were compared using independent‐samples *t* tests. Categorical variables were compared using Fisher's exact tests. Regarding the type of surgery, statistical comparisons were restricted to the total cohort, as subgroup analyses by graft type resulted in insufficient cell counts for valid inferential testing.

After testing for parametric distribution using the Shapiro–Wilk test and graphic testing, non‐parametric tests were indicated for further comparison between the groups. Therefore, Mann–Whitney *U* test was used for testing between male and female patients. As two primary outcomes were defined a priori (the maximum knee valgus angle during the drop jump and during the single leg jump), Holm's step‐down correction was applied to control the family‐wise error rate for these two predefined primary hypotheses. All additional biomechanical variables were considered secondary and exploratory. Therefore, no multiplicity correction was applied to these measures; instead, unadjusted *p* values together with effect sizes (Cohen's *d*) are reported to allow transparent interpretation of the results.

When interpreting the knee joint angles in the frontal plane in this study, it should be noted that negative values always represent valgus values and positive values always represent varus values.

## RESULTS

Demographic data for the included patients are shown in Table 1.

**Table 1:** Characteristics of included patients (average ± standard deviation, minimum and maximum).

	Male	Female	*p*
Age	24.8 ± 4.8 (18–33 years)	26.2 ± 6.9 (18–40 years)	0.596
BMI	24.8 ± 3.6 (20.8–33.2)	24.8 ± 5.1 (18.3–35.7)	0.987
Surgery			
ST‐autograft	*n* = 8	*n* = 7	0.217
QT‐autograft	*n* = 9	*n* = 2	
Total	*n* = 17	*n* = 9	
Follow‐up (months)	9.3 ± 3.1	8.8 ± 1.8	0.595

Abbreviations: BMI, body mass index; QT, quadriceps tendon; ST, semitendinosus tendon.

There was no significant difference regarding age, body mass index (BMI) and time until follow‐up between male and female participants (see Table 1).

## VERTICAL DROP JUMP

**Figure 3:**
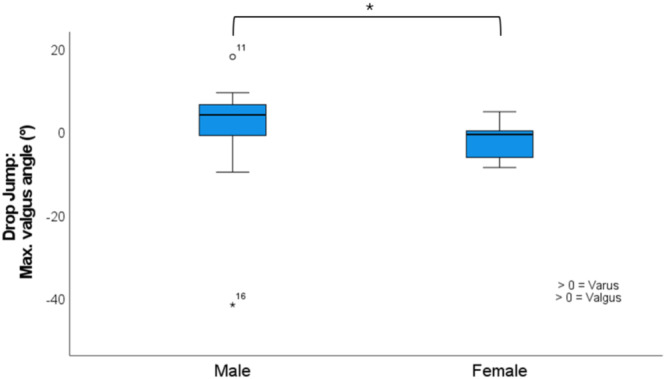
Boxplot illustrating the maximal knee valgus angle during the vertical drop jump test in male and female participants. Group differences were analysed using the Mann–Whitney *U* test. *p < 0.05.

In the vertical drop jump test, the median of the maximum valgus angle (primary outcome measure) was −2.9° in the female group compared to 6.7° in the male group (see Figure 3). This was a significant difference (*p* = 0.016, adjusted *p* = 0.032).

There was no significant difference between male and female patients regarding maximum valgus moment, maximum knee flexion angle and maximum hip flexion angle (see Table 2).

**Table 2:** Results of the vertical drop jump test in men and women, median (IQR).

**Parameter**	**Group**	**Median (IQR)**	***p***	**Cohen's *d***
Maximum knee flexion angle (°)	Men	67.2 (61.7–76.9)	0.367	0.378
	Women	67.0 (53.1–71.8)		
Maximum knee valgus angle (°)	Men	6.7 (−0.2 to 8.5)	0.016*	0.491
	Women	−2.9 (−9.8 to 2.0)	Adj.: 0.032*,^a^	
Maximum hip flexion angle (°)	Men	60.4 (52.4–66.9)	0.241	0.434
	Women	53.6 (47.0–62.3)		
Maximum knee valgus moment (N·mm/kg)	Men	−68 (−169 to −13)	0.672	0.389
	Women	−69 (−120 to −5)		

*Note*: Maximal knee abduction angle < 0 represents valgus angles, > 0 represents varus angles.

Abbreviations: Adj., adjusted; IQR, interquartile range.

a Adjusted *p* (Holm's step‐down correction).

* *p* < 0.05.

### Single‐leg jump for distance

**Figure 4:**
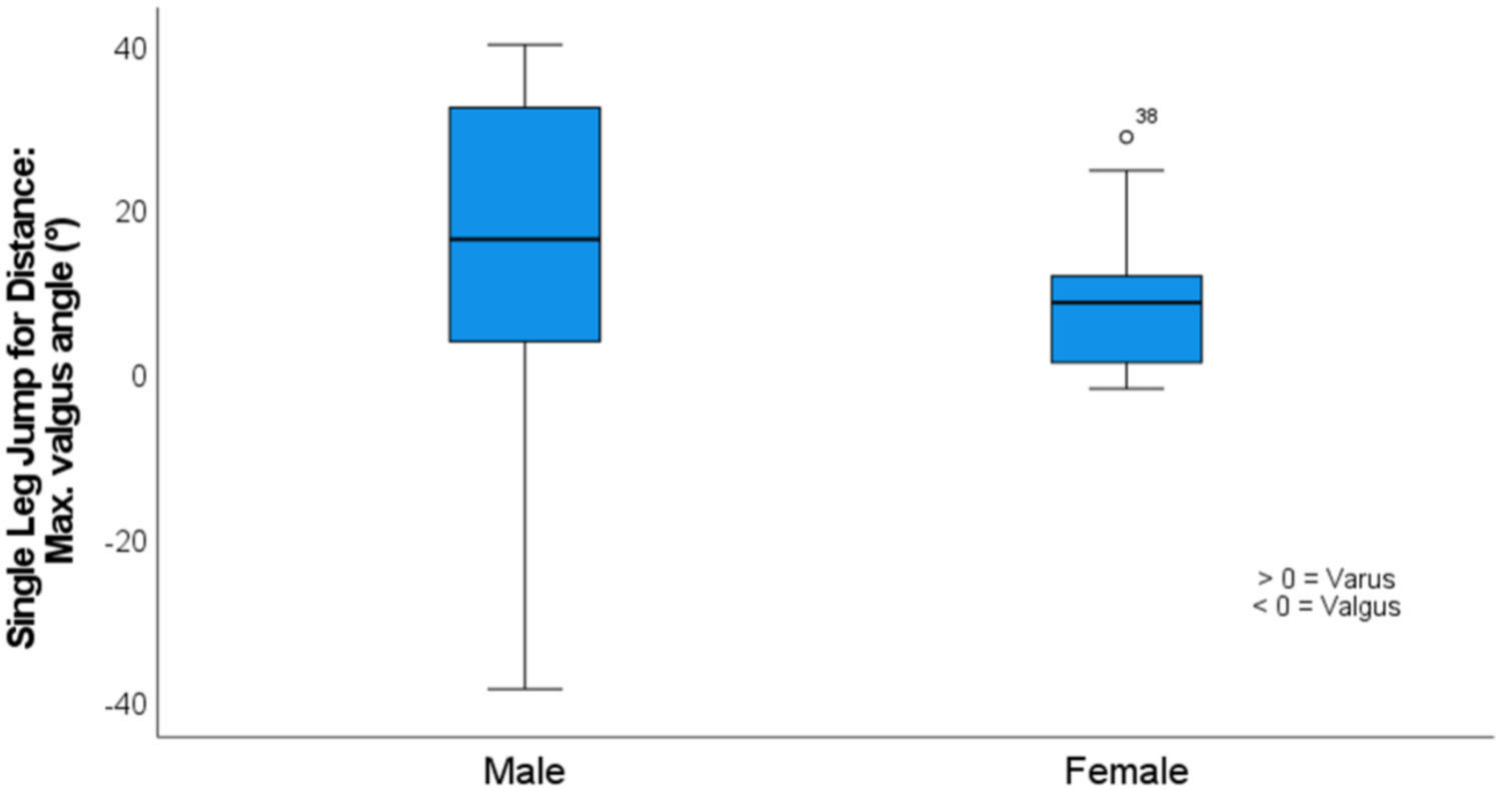
Boxplot illustrating the maximal knee valgus angle during the single‐leg jump for distance in male and female participants. Group differences were analysed using the Mann–Whitney *U* test.

In the single‐legged jump test, there was no significant difference regarding the maximal knee valgus angle, maximal knee and hip flexion angle between male and female patients (see Table 3 and Figure 4).

**Table 3:** Results of the single‐leg jump for distance in men and women, median (IQR).

Parameter	Group	Median (IQR)	*p*	Cohen's *d*
Maximum knee flexion angle (°)	Men	57.8 (46.8–70.3)	0.958	0.002
	Women	62.9 (50.0–68.9)		
Maximum knee valgus angle (°)	Men	16.2 (0.9–33.3)	0.312	0.201
	Women	8.5 (−0.3 to −18.1)	Adj.: 0.312*,^a^	
Maximum hip flexion angle (°)	Men	58.8 (52.0–75.5)	0.711	0.304
	Women	57.8 (52.8–63.2)		

*Note*: Maximal knee abduction angle < 0 represents valgus angles, > 0 represents varus angles.

Abbreviations: Adj., adjusted; IQR, interquartile range.

a Adjusted *p* (Holm's step‐down correction).

* *p* < 0.05.

### Isokinetic torque

No significant difference was observed between male and female patients regarding the H/Q ratio (median 0.48 [interquartile range, IQR 0.45–0.50] vs. 0.45 [IQR 0.40–0.47]; *p* = 0.051, Cohen's *d* = 0.790).

## DISCUSSION

The main finding of this study is that women tended to land with a higher valgus angle in the contralateral knee after ACL reconstruction than men. This confirms the hypothesis.

The moderate effect size observed for the valgus angle in the drop jump supports the relevance of this primary finding. In contrast, the secondary parameters demonstrated only small to moderate effect sizes, and these differences did not reach statistical significance. Therefore, no clear conclusions can be drawn regarding potential sex differences in these secondary outcomes. Whether the observed tendencies reflect true biomechanical differences or are rather due to limited statistical power remains unclear.

This assumption was plausible, as repeatedly demonstrated in the literature, women have a higher risk of primary and secondary ACL injuries [45, 47]. One key explanation for this is the greater incidence of dynamic valgus movements in female athletes [26]. Since an increased valgus angle is known as an important biomechanical risk factor for ACL injuries [17], it was reasonable to expect the valgus angles in women to be greater in the present cohort.

The results of this study support this assumption: in the drop jump, the valgus angle was on average 9.6° higher in women, which was statistically significant. In the single‐leg jump, the difference was similar at 7.7°, but did not reach statistical significance.

Several studies have demonstrated that dynamic valgus in female athletes can be effectively reduced through targeted neuromuscular training programmes, such as the FIFA 11 + injury prevention programme or the STOP‐X programme [13, 38, 40].

The following mechanisms are cited in the literature as neuromuscular causes of valgus collapse of the knee: insufficient activation and control of the hip abductors and external rotators (especially the gluteus medius and maximus muscles [39]), dominant activation of the quadriceps muscles with simultaneous weak hamstring activity and deficits in trunk stability [16]. Consequently, interventions focusing on hip and trunk stabilization, neuromuscular control and landing mechanics have shown significant improvements in valgus alignment during jump‐landing tasks [13, 38, 40]. These findings indicate that dynamic valgus is a relevant modifiable biomechanical risk factor. This highlights the potential for structured prevention and rehabilitation programmes to reduce valgus stress, particularly in female athletes. Clinically, this implies that therapists should systematically assess valgus control and apply individualized neuromuscular training programmes in rehabilitation after ACL replacement. Such targeted programmes may help reduce contralateral ACL injury risk in female athletes returning to sport [16].

Recent international rehabilitation guidelines support these clinical implications. The formal EU–US Meniscus Rehabilitation 2024 Consensus [37] highlights the importance of criterion‐based and individualized neuromuscular training as a central element of postoperative care. Although primarily developed for meniscus surgery, the consensus emphasizes neuromuscular control of the lower limb as a key modifiable factor across knee pathologies, particularly in cases of combined injuries such as ACL tears with concomitant meniscal damage—an injury pattern seen in more than 50% of ACL ruptures [41].

Other studies have also demonstrated gender‐specific differences in biomechanics following ACL injury and reconstruction. For example, Asaeda et al. [3] showed that women exhibited increased tibial external rotation compared to men, which persisted even after ACL reconstruction. These differences in rotational kinematics during the stance phase of gait illustrate that gender‐specific differences manifest not only in dynamic tests such as jump landings but also in cyclic movement patterns.

In addition, Lefebvre et al. [27] reported gender‐specific adaptations of gait patterns in ACL deficiency in a retrospective analysis. While men primarily exhibited reduced varus position and increased external rotation, women showed significantly more pronounced adaptations in the sagittal and frontal planes, particularly an increased valgus angle over large parts of the gait cycle. These findings are consistent with the results of the present study, which document a larger valgus angle in women when landing. They support the assumption that the valgus angle could be a gender‐specific risk factor for re‐injury.

Regarding the median H/Q ratio, there was no significant difference between men (0.48) and women (0.45). This is consistent with the literature, which reports only minor differences between the sexes at low test velocities [18, 25]. Nevertheless, the large effect size in this study suggests that a relevant difference cannot be excluded. As lower hamstring strength may reduce control of anterior tibial translation and valgus loading in landing tasks [8, 46], a lower H/Q ratio in female athletes could contribute to the observed movement patterns. While our data cannot confirm this relationship statistically, the trend is biomechanically plausible and may warrant further investigation in studies with larger sample sizes.

Overall, the values in this cohort are at the lower end of the range reported in the literature [4, 18, 23]. One possible explanation for this could also be the relatively low test velocity in the present study of 60°/s, as the H/Q ratio increases at higher test speeds [4, 18].

In summary, this study has shown that women following ACL reconstruction tend to engage in movement patterns that pose a risk for the non‐operated ACL more than men at a time when many athletes are returning to sport. This period is particularly relevant, as the risk for secondary ACL injury is 15 times higher in the first 12 months after ACL reconstruction (ACLR) than in a previously uninjured cohort [33]. A prospective case‐control study by Paterno et al. also showed that female athletes had a 6 times higher risk for a contralateral ACL injury than male athletes in this time period [33]. Clinically, these findings could be used to identify athletes at risk and for improved and individualized rehabilitation and prevention.

A significant limitation of this study is the lower number of women included compared to the initial sample calculation. This reduces the statistical power to reliably detect gender‐specific differences and increases the risk of a β‐error. The total number of cases also remained below the target size, which limits the generalizability of the results. Furthermore, this precludes the meaningful application of more complex statistical methods, such as multivariate regression. Given the underpowered sample, the findings of this study should be considered exploratory.

Since the movement analyses were performed under standardized laboratory conditions, it also remains unclear to what extent the results can be transferred to real game situations.

As this investigation is a secondary analysis of a previously published cohort, the results are based on existing data and the predetermined sample composition. However, all patients were treated at the same clinical centre, operated on by the same surgeon and followed an identical rehabilitation protocol, minimizing potential centre‐ or protocol‐related biases. Since the present analysis focuses only on the contralateral, non‐operated limb, the influence of graft‐specific surgical differences (semitendinosus vs. quadriceps tendon graft) on the current outcomes is expected to be minimal.

As only two primary outcomes were defined a priori, multiplicity was controlled using Holm's procedure. Secondary analyses were exploratory and are presented without adjustment for multiple testing according to established recommendations [6, 43]. These results should therefore be interpreted cautiously, with effect sizes provided to support transparent evaluation of potential trends.

A further limitation is that the present study cannot establish a direct association between the identified biomechanical patterns and actual ACL re‐injury risk, as no longitudinal follow‐up was performed. The findings should therefore be interpreted as indicators of potential risk rather than predictors of confirmed injury. Future prospective studies with systematic follow‐up are required to determine whether these sex‐specific movement patterns translate into different re‐injury rates.

Despite these limitations, we are convinced that this study provides useful information for the detection of athletes at risk and thus contributes to individualized injury prevention. In training and physical therapy, dynamic valgus should be a particular focus, especially for female athletes who have already suffered an ACL rupture.

## CONCLUSION

Female patients demonstrated greater contralateral dynamic valgus during drop jump landings after ACL reconstruction, indicating sex‐specific neuromuscular risk patterns. These results highlight the relevance of individualized rehabilitation and secondary prevention strategies, with particular emphasis on addressing dynamic valgus in female athletes following ACL reconstruction.

## AUTHOR CONTRIBUTIONS

Sebastian Bierke, Tilman Hees, Kerstin Siemßen, Bernd Wolfarth and Wolf Petersen contributed to the study conception and design. Data collection and analysis were performed by Fredrik Gaugg, Tilman Hees and Sebastian Bierke. The first draft of the manuscript including figures and tables was written by Fredrik Gaugg. All authors commented on previous versions of the manuscript. All authors read and approved the final manuscript.

## CONFLICT OF INTEREST STATEMENT

Wolf Petersen reports royalties or licences from Karl Storz (Tuttlingen, Germany); consulting fees from OPED (Valley, Germany) and Arthrex (Munich, Germany); payment or honoraria for lectures, presentations, manuscript writing or educational events from Plasmaconcept (Cologne, Germany), Arthrex (Munich, Germany), OPED (Valley, Germany) and Otto Bock (Duderstadt, Germany); leadership or fiduciary role in other board, society, committee or advocacy group (unpaid); and roles as Vice President of GOTS (Society of Orthopedics and Trauma in Sports) and Board Member of the German Knee Society (DKG). The remaining authors declare no conflicts of interest.

## ETHICS STATEMENT

The study was approved by the Ethics Committee of the Humboldt University of Berlin (HU‐KSBF‐EK_2019_0016). The study was conducted in accordance with the Declaration of Helsinki. All participants were informed about the purpose and procedures of the study and provided both verbal and written informed consent prior to participation.

## Supporting information

STROBE_checklist_DynValg.

## Data Availability

The data that support the findings of this study are available from the corresponding author upon reasonable request.
